# Genetic differences between Asian and Caucasian chronic lymphocytic leukemia

**DOI:** 10.3892/ijo.2013.1966

**Published:** 2013-05-27

**Authors:** NORIHIKO KAWAMATA, CHIMENE MOREILHON, TAKAYUKI SAITOH, MASAMITSU KARASAWA, BRIAN K. BERNSTEIN, AIKO SATO-OTSUBO, SEISHI OGAWA, SOPHIE RAYNAUD, H. PHILLIP KOEFFLER

**Affiliations:** 1 Hematology/Oncology, Cedars-Sinai Medical Center, Los Angeles, CA, USA;; 2 Center Hospital, University of Nice, Nice, France;; 3 Gunma University, Maebashi, Gunma, Tokyo, Japan;; 4 University of Tokyo, Tokyo, Japan;; 5 National University of Singapore, Singapore

**Keywords:** SNP-chip, common genomic changes, caspase, IRF4

## Abstract

Chronic lymphocytic leukemia (CLL) is a common hematological malignancy in Western countries. However, this disease is very rare in Asian countries. It is not clear whether the mechanisms of development of CLL in Caucasians and Asians are the same. We compared genetic abnormalities in Asian and Caucasian CLL using 250k GeneChip arrays. Both Asian and Caucasian CLL had four common genetic abnormalities: deletion of 13q14.3, trisomy 12, abnormalities of ATM (11q) and abnormalities of 17p. Interestingly, trisomy 12 and deletion of 13q14.3 were mutually exclusive in both groups. We also found that deletions of miR 34b/34c (11q), caspase 1/4/5 (11q), Rb1 (13q) and DLC1 (8p) are common in both ethnic groups. Asian CLL more frequently had gain of 3q and 18q. These suggest that classic genomic changes in the Asian and Caucasina CLL are same. Further, we found amplification of IRF4 and deletion of the SP140/SP100 genes; these genes have been reported as CLL-associated genes by previous genome-wide-association study. We have found classic genomic abnormalities in Asian CLL as well as novel genomic alteration in CLL.

## Introduction

Development of cancers are affected by a number of factors including environment and genetic background (1). Difference in incidence of certain cancers between Asian and Caucasian populations is well-recognized (2,3). Environmental and/or genetic factors may contribute to these differences. For example, a high incidence of gastric cancers have been reported in Asian countries including Japan (3,4). However, the incidence of gastric cancers in Japanese immigrants to Hawaii is much lower than the frequency in Japanese in their homeland (4,5). This high incidence of gastric cancer appears to be strongly influenced by environmental factors. In contrast, Asian non-smoking women have a high incidence of non-small cell lung cancer (NSCLC) associated with an EGFR mutation irrespective of the country where they live (6,7), suggesting a germline predisposition for NSCLC with this mutation.

Chronic lymphocytic leukemia (CLL) is a common hematological malignancy in Western countries and it is very rare in Asian countries, including Japan (8–10). Furthermore, Asians including Japanese immigrants to USA continue to have a low incidence of CLL (11,12).

This difference in incidence of CLL between Asians and Caucasians is not clear. One possible explanation for the difference may be that CLL in these two distinct ethnic groups show two distinctly different molecular signatures. Another possible explanation may be that environmental factors, for example an infectious disease, could cause the development of CLL and affect the incidence of this disease in two geographically distinct regions. This seems less likely because Asians in USA also have a low incidence of CLL (11,12). A third possibility is germline variation in the general population of these two ethnic groups leads to the difference in the incidence of the development of CLL.

To identify molecular signatures of Caucasian and Asian CLL at the DNA level, we analyzed these cells using high resolution single nucleotide polymorphism (SNP) genomic oligonucleotide microarrays.

## Materials and methods

### Samples

Diagnosis of CLL was made based on the updated National Cancer Institute-Working Group guidelines (13). All leukemic cells expressed CD5, CD19 and CD20. Rearrangement of the immunoglobulin heavy chain gene was confirmed by PCR as described previously (14). Detailed information of the Japanese CLL has been reported previously (14). Leukemic cells were collected from 77 cases of Asian CLL (75 Japanese and 2 Chinese) and 55 Caucasian CLL after their informed consents were obtained. Institutional committees approved the experiments undertaken. DNA was extracted using Qiagen DNA extraction kit according to the manufactuer’s protocol.

### SNP-chip analysis

The extracted DNAs were digested with Nsp-I restriction enzyme, amplified and hybridization with GeneChip250NspI chip from Affymetrix. Hybridized signals were captured on GeneChip Scanner 3000 and the data were analyzed by CNAG3.3 software (15,16). Significant differences were analyzed using χ^2^ tests. P-values <0.05 were considered statistically significant.

## Results

### Caucasian and Asian CLL shared the major genomic copy number changes

We analyzed 55 cases of Caucasian CLL and 77 cases of Asian CLL and found a number of genomic abnormalities in both groups. The four well characterized genomic abnormalities of CLL, including deletion of 13q14, trisomy 12, deletion/UPD of 17p and deletion/UPD of 11q (ATM) (17,18), were detected in both groups at a comparable frequency except for deletion/UPD of 11q. Asian v/s Caucasian CLL had deletion of 13q14.3 (Asian: 39 cases, 51%; Caucasian: 26 cases, 48%), trisomy 12 (Asian: 15 cases, 20%; Caucasian: 16 cases, 30%), abnormalities of 11q (Asian: 21 cases, 27%; Caucasian: 5 cases, 10%) and abnormalities of 17p (Asian: 11 cases, 15%; Caucasian: 3 cases, 5%) (Table I). Only the frequency of 11q was statistically different (P<0.01). Interestingly, deletion of 13q14 and trisomy 12 was mutually exclusive in Asian CLL cases (no cases with both trisomy 12 and 13q14 in the 77 Asian CLL) and nearly same in Caucasian CLL (2 of the 55).

Other genomic alterations involved four regions: trisomy 3/duplication of 3q; trisomy 18/duplication of 18q; deletion of 18p; and deletion of 8p (Table II). Asians more frequently had either trisomy 3/duplication (dup) of 3q or trisomy 18/dup18q compared to the Caucasian CLL samples (18 Asian v.s. 0 Caucasian CLL sample) (Table II).

### Novel candidate target genes associated with development of CLL

**Figure 1:**
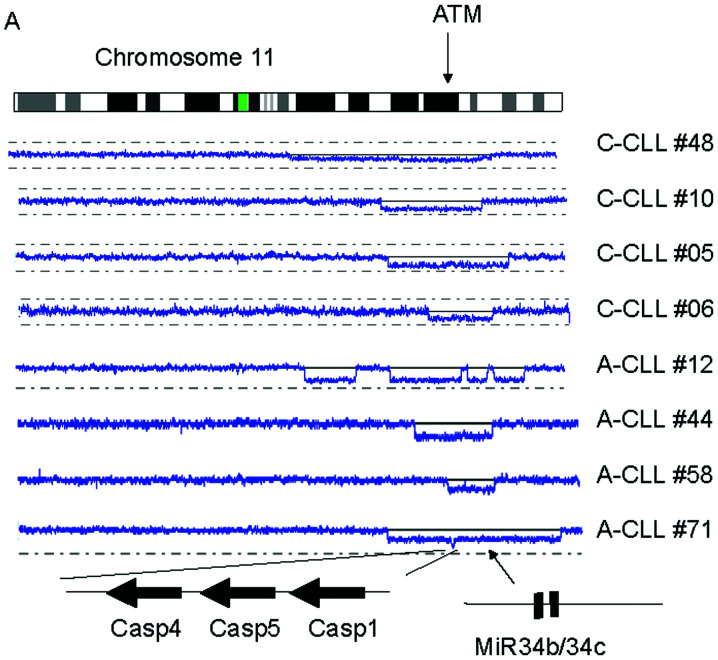

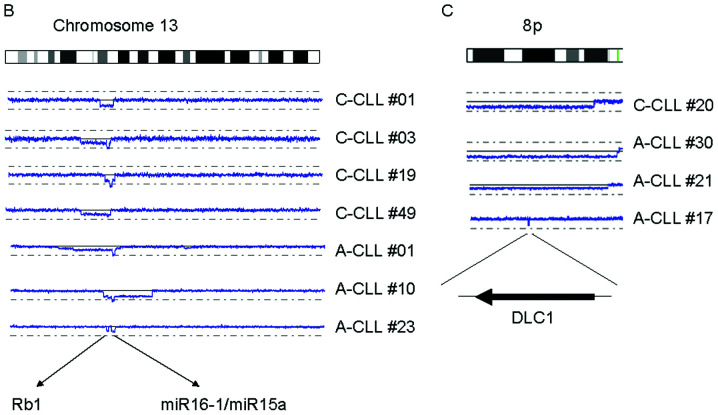
Novel common target candidate genes in CLL. (A) Representative CLL cases with deletion of 11q. SNP-chip analysis (blue lines) is shown with a scheme of the chromosome (top), individual CLL samples and their altered genes (bottom). The arrow on the chromosome panel indicates the position of the ATM gene. At the bottom, the three large arrows show the direction of transcription of the target genes; and the diagonal arrow designates the position of the microRNAs, miR 34b/34c. The CLL case numbers are shown on the right side (A, Asian; C, Caucasian). (B) Representative CLL cases with deletion of 13q. The results of SNP-chip analysis (blue lines) are shown together with the scheme of the chromosome (top). Arrows at the bottom indicate the positions of RB1 gene and the microRNAs, miR 15-a/16-1. The case numbers are shown on the right side (A, Asian; C, Caucasian). (C) Representative CLL cases with deletion of 8p. The result of SNP-chip analysis (blue lines) is shown with the scheme of the chromosome (top) and genes (bottom). The arrow indicates the direction of transcription of the DLC1 gene. The case numbers are shown on the right side (A, Asian; C, Caucasian).

In this study, we found novel tumor suppressor candidate genes often deleted in CLL. Deletion of 11q is frequently detected in CLL, involving the ATM gene (26/132 cases in total); however, of interest, these 26 cases also had deletion of the microRNA (miR) 34b/34c (Fig. 1A and data not shown). A second novel finding was a homozygous deletion of 11q22.3 involving caspase 1/4/5 genes in an Asian CLL sample (Fig. 1A); and seven additional cases had hemizygous deletion of these caspase genes (Fig. 1A).

The third finding was near the well-known commonly deleted region at 13q14.3 (65 cases in total) involving miR 15-a and 16-1 (Table I and Fig. 1B). One case had two distinct deletions in this region; one involved miR 15-a and 16-1; and the other encompassed the Rb1 gene (Asian CLL #23 in Fig. 1B). On close inspection, 36% of cases with deletion of 13q (23/65 cases), had a deletion of Rb1, suggesting that Rb1 might be another target of deletion of 13q in CLL.

Fourth, we found that deletion of 8p was common in CLL (5 cases) (Table II and Fig. 1C). One Asian-CLL had a very small deletion only spanning the DLC1 gene (Asian-CLL #17, Fig. 1C). The DLC1 gene may be one of the target genes in those samples with deletion of 8p in CLL.

### CLL-associated genes are genomically abnormal in CLL samples

**Figure 2:**
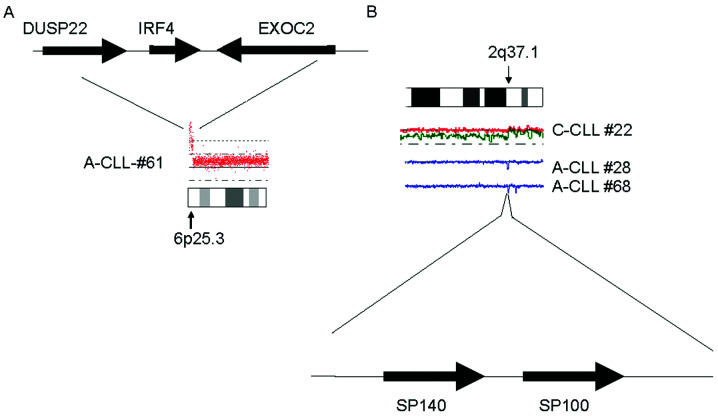
Genes close to high-risk SNP sites are targets for genomic alterations in CLL. (A) CLL with amplification of IRF4. The result of SNP-chip analysis (orange dots) is shown with the scheme of the chromosome (bottom) and genes (top). An arrow on the chromosome panel shows the position of the 6p25.3 involving the IRF4 gene. Arrows on the top panel indicate the direction of transcription of the genes. The case number is shown on the left side (A, Asian). (B) CLL cases with deletion of SP140. The result of SNP-chip analysis (blue lines or red/green lines) is shown with the scheme of the chromosome (top). An arrow on the chromosome panel indicates the position of 2q37.1 involving SP140. Arrows and their diagonal lines at the bottom indicate the direction of transcription and location of SP100/SP140 genes. The case numbers are shown on the right side (A, Asian; C, Caucasian).

One Asian-CLL sample had high copy number amplification of IRF4 (Fig. 2A). Also, we found that SP140/SP100 genes were deleted in two Asian and one Caucasian CLL samples (Fig. 2B).

A previous genome-wide association study (GWAS) identifies SNP sites related to Caucasian CLL (19), including SNP-rs872071 (6p25.3) located close to IRF4 gene and SNP-rs13397985 (2q37.1) located close to the SP140/SP100 genes.

## Discussion

CLL is extremely rare in Asians (10–12). We have for the first time, analyzed a large number of Asian CLL samples, using high resolution SNP-chips. Well-known common genomic abnormalities (17,18) occured in Asian CLL at a comparable frequency as Caucasian CLL except for deletion/UPD of 11q. Thus, the classic genomic abnormalities of CLL in these two ethnic groups are similar at the DNA level. However, the Asian CLL had trisomy 3/duplication of 3q and trisomy 18/duplication of 18q more frequently than Caucasian CLL samples, suggesting that the mechanism of development of CLL in Asians is slightly different from Caucasian CLL.

The 18q region contains the BCL2 gene. BCL2 is associated with an anti-apoptotic effect and the gene is often overexpressed in CLL (20,21). CLL-related loss of 13q14, deletes miR15-a/miR 16-1 (22–24). These two microRNAs target BCL2 and their deletion causes high expression of BCL2 (24,25). Trisomy 18/duplication of 18q may be an alternative mechanism to overexpress BCL2. Also, the 3q region contains BCL6 (26,27), whose protein antagonizes the TP53 tumor suppressor gene (27,28).

Seven cases had an 8p deletion (3/77 Asian CLL, 1/55 Caucasian CLL); and the minimally commonly deleted region involved DLC1. In fact, one sample (Asian-CLL 17) had a deletion that only involved the DLC1 gene. This gene encodes the GAP protein and plays an important role in the RAS/MAP pathway in a manner similar to NF1 (29,30). This gene is frequently deleted in a variety of cancers (30–33). Our data suggest that dysregulation of RAS/MAP signal pathway occurs in CLL. Reagents targeting this signaling pathway could be a promising treatment option for these CLL cases.

Deletion of 8p, duplication of 3q and duplication of 18q are also frequently detected in other types of B-cell malignancies including lymphomas (34,35). Although a variety of B-cell lymphoid malignancies have their distinct clinical features (36), they may have dysregulation of common signaling pathways. Identification of molecular signatures by SNP-chip may lead to re-classification of B-cell malignancies and guide clinicians to select optimal therapeutic options targeting the dysregulated signaling pathways.

In this study, we also found novel candidate target genes in altered chromosomal regions, which may be involved in development of CLL. We found that Rb1 on the 13q chromo-some is frequently deleted when 13q14 is deleted (Fig. 1B). This was especially evident in the sample, A-CLL #23, which had two distinct deletions in 13q; one targeting 13q14 (miR15-a/miR16-1) and the other targeting RB1. Thus, Rb1 may be another tumor suppressor gene on 13q that is altered in CLL. In addition, a homozygous deletion of three gene family (Casp1/4/5) occurred at 11q in A-CLL #71. Both ATM and miR34b/34c genes are on 11q and are frequently deleted in CLL. A short distance away are these three caspase genes which can also be target genes of this common deleted regions. Casp1/4/5 are components of the inflamasome associated with inflamation and cell growth (37,38). Deletion of these genes may cause an abnormal response of leukemic cells to the immuno-surveillance system, allowing proliferation of the leukemic cells.

GWAS co-relates presence or absence of a disease with the patterns of SNPs in large populations, leading to identification of high-risk alleles strongly associated with development of the disease (39,40). Interestingly, the genes close to these CLL-associated SNP-sites (IRF4 and SP140/SP100) are moderately often mutated (deleted/amplified) in CLL.

In conclusion, we found that: i) the fundamental molecular signatures of CLL in the Caucasian and Asian populations are fairly similar; ii) novel candidate target genes in commonly altered genomic regions include Rb1, Casp1/4/5, DLC1, IRF4 and SP140/SP100; iii) CLL-associated genes identified by the previous GWAS were moderately often mutated in CLL. These findings will help to re-classify CLL based on their molecular signatures and lead to individualized treatment for CLL with reagents targeting signaling pathways which are altered in each patient.

## Figures and Tables

**Table I:** Four common genomic abnormalities in Asian and Caucasian CLL.

	Asian CLL (77)	Caucasian CLL (55)	P-value
13q14 deletion	39 (51%)	26 (48%)	0.60
(sole 13q14 deletion)	12 (16%)	8 (15%)	0.66
11q deletion/UPD	21 (27%)	5 (10%)	<0.01^a^
17p deletion/UPD	11 (15%)	3 (5%)	0.06
Trisomy 12	15 (20%)	16 (30%)	0.09

Seventy-seven Asian and 55 Caucasian samples of CLL were examined with SNP-chip. Four common genomic abnormalities are tabulated. The number of cases with indicated abnormalities are shown. Sole 13q14 deletion indicates the numbers of cases with only 13q14 deletion genomic abnormality noted on SNP-chip analysis. P-values are also shown.

a Statistical siginificance.

**Table II:** Other common genomic abnormalities found in Asian and Caucasian CLL.

	Asian CLL (77)	Caucasian CLL (55)	P-value
Trisomy 3/Dup 3q	6 (8%)	0 (0%)	<0.01^a^
Trisomy 18/Dup 18q	8 (11%)	0 (0%)	<0.01^a^
Del 8p	4 (7%)	1 (2%)	0.11
Del 18p	3 (4%)	3 (5%)	0.47

The number of cases with indicated abnormalities are shown. P-values are also shown.

a Statistical significance.
